# Chloroplast DNA Phylogeography Reveals Repeated Range Expansion in a Widespread Aquatic Herb *Hippuris vulgaris* in the Qinghai-Tibetan Plateau and Adjacent Areas

**DOI:** 10.1371/journal.pone.0060948

**Published:** 2013-04-02

**Authors:** Jin-Ming Chen, Zhi-Yuan Du, Shan-Shan Sun, Robert Wahiti Gituru, Qing-Feng Wang

**Affiliations:** 1 Key Laboratory of Aquatic Botany and Watershed Ecology, Wuhan Botanical Garden, The Chinese Academy of Sciences, Wuhan, Hubei, China; 2 Graduate University of the Chinese Academy of Sciences, Beijing, China; 3 Botany Department, Jomo Kenyatta University of Agriculture and Technology, Nairobi, Kenya; University of Lausanne, Switzerland

## Abstract

**Background:**

The Qinghai-Tibetan Plateau (QTP) is one of the most extensive habitats for alpine plants in the world. Climatic oscillations during the Quaternary ice age had a dramatic effect on species ranges on the QTP and the adjacent areas. However, how the distribution ranges of aquatic plant species shifted on the QTP in response to Quaternary climatic changes remains almost unknown.

**Methodology and Principal Findings:**

We studied the phylogeography and demographic history of the widespread aquatic herb *Hippuris vulgaris* from the QTP and adjacent areas. Our sampling included 385 individuals from 47 natural populations of *H*. *vulgaris*. Using sequences from four chloroplast DNA (cpDNA) non-coding regions, we distinguished eight different cpDNA haplotypes. From the cpDNA variation in *H*. *vulgaris*, we found a very high level of population differentiation (*G*_ST_ = 0.819) but the phylogeographical structure remained obscure (*N*_ST_ = 0.853>*G*_ST_ = 0.819, *P*>0.05). Phylogenetic analyses revealed two main cpDNA haplotype lineages. The split between these two haplotype groups can be dated back to the mid-to-late Pleistocene (ca. 0.480 Myr). Mismatch distribution analyses showed that each of these had experienced a recent range expansion. These two expansions (ca. 0.12 and 0.17 Myr) might have begun from the different refugees before the Last Glacial Maximum (LGM).

**Conclusions/Significance:**

This study initiates a research on the phylogeography of aquatic herbs in the QTP and for the first time sheds light on the response of an alpine aquatic seed plant species in the QTP to Quaternary climate oscillations.

## Introduction

Profound climatic oscillations during the Quaternary ice age of the past ∼2 million years have left imprints on modern-day biota by effecting species' distributions, evolution and extinction [1]–[5]. Such range and demographic changes undoubtedly affect the geographic genetic structure of species [4]. Molecular-based phylogeographical methods have been widely used to unravel the evolutionary history of species by tracing the genetic footprints of Quaternary climatic shifts in different regions, particularly in Europe, North America, and Japan [5]–[7]. Numerous phylogeographical surveys in Europe, North America, and Japan have identified the locations of glacial refugia and routes of colonization/range expansion of organisms after glacial periods in these regions [5]. In contrast, knowledge of phylogeographical histories of organisms occurring in mainland China as well as the association of this history with climatic fluctuations, is relatively limited, particularly for plants [5], [8]. Such information would clarify why China is such an important center of global biodiversity [9], [10].

The Qinghai-Tibetan Plateau (QTP) located in western China is the highest and largest plateau on earth, with an average altitude of approximately 4,500 m and an area of 2.5×10^6^ km^2^ [11]. The phased uplift of the QTP, which probably started with the collision of Indian and Eurasian plates about 50 million years ago [12]–[15], has greatly changed the geology and topography of East Asia, resulting in the significant climatic and environmental changes witnessed since the early Cenozoic Era [16]–[18]. The QTP has experienced several glacial and interglacial cycles since the Pliocene [19], [20]. The recent uplifts of much of the QTP during the mid-to-late Quaternary may have helped to drive the onset of mountain glaciers, ice caps, and valley glaciers [20]–[23]. However, it is unlikely that a unified ice sheet developed on the QTP in the late Pleistocene and Quaternary periods [23], [24]. Although no unified massive ice sheet could have developed in the QTP during glacial periods [23], [24], the alternating glacial and interglacial periods have strongly and periodically influenced the environments and vegetation of the QTP [24], [25].

It is estimated that the present-day alpine flora of the QTP (above 4,000 m) numbers *ca*.1,816 seed plant species in more than 339 genera, with *ca*.33 genera being endemic [26]. The Hengduan Mountains (HDM, reaching 7,556 m), located at the southeastern edge of the QTP [27], represent one of the world's biodiversity hotspots [28]. The vegetation in the QTP and adjacent highlands is considered to be highly sensitive and vulnerable to global climate change because plant growth and distribution in the region depend greatly on survivable temperatures [11]. The intensity of the uplift of QTP in the late Pliocene combined with repeated climate fluctuations during the Quaternary have dramatically influenced the distribution and evolution of many plant species in these regions [22], [26]. However, how the distribution ranges of plant species shifted on the QTP in response to Quaternary climatic changes is still in question. Recent phylogeographical studies have indicated remarkable differences in the demographic history of the plant species from the QTP. Some studies provide evidence favoring the hypothesis that the extant plant populations on the plateau are colonists from the low altitude refugia at the plateau edge (e.g., the HDM) during the Last Glacial Maximum (LGM) and /or previous glacial periods [29]–[32]. In contrast, several other studies support the hypothesis that species probably survived on high-altitude parts of the central Plateau throughout the Quaternary [33]–[39]. Alternatively, additional studies have indicated that the tectonic events rather than the Quaternary climatic oscillation may have played the most important role in plant divergence and speciation in the QTP [10], [40]–[42]. Species with different ecological requirements and distributions may have led to dissimilarities in refugia and migration routes [43]. The current plant phylogeographical studies on the QTP have left large gaps in taxonomic and habitat sampling; for example, as yet there are only few published data examining phylogeographical patterns on herbaceous plants [31]–[33], [38], [39], [42], [44], with no research on aquatic or wetland plant phylogeography.

In the QTP and adjacent areas, the large number of high altitude lakes, ponds, streams, rivers and wetlands [45] support *ca*.133 aquatic herbaceous seed plants species in 63 genera and 29 families [46]. Eleven of these species are endemic to China but a few (four species, e.g., *Puccinellia degeensis*, *P*. *multiflora*, *Carex sagaensis*, *C*. *muliensis*) are endemic to the QTP and adjacent areas [46]. Species richness of the aquatic plants exhibits a tendency whereby there is gradual decrease in total species number from the southeast edge of the QTP (e.g., the HDM) to the northwest region [46]. In the northern region, the flora of aquatic plants comprises only a few widespread and tolerant species [46]. Aquatic plants typically have much broader geographical distributions and ranges compared to their terrestrial counterparts [47], [48]. As opposed to trees, the range shifts of herbaceous aquatic plants could have responded more quickly to climate or vicariance [48], [49]. However, for aquatic plants in the QTP and adjacent regions, patterns of range shifts following climate change or vicariance are poorly understood.

In this study, we assessed the phylogeographical pattern of an aquatic perennial herb, *Hippuris vulgaris* (Hippuridaceae), a species with a worldwide distribution, in the QTP and adjacent areas. In China, the species is mainly distributed in the Southwest, Northeast and Northwestern regions [50]. The plant grows in ponds, ditches, shallow riverbeds, and other freshwater wetlands. *Hippuris vulgaris* is a major and widespread component of aquatic vegetation in the QTP and adjacent areas. It can be found at altitudes of up to 5,000 m and is a highly cold tolerant species [46]. We investigated the variations in chloroplast DNA (cpDNA) non-coding regions (*ycf*6-*psb*M, *trn*T-*trn*L, the *rps*16 intron and *atp*I-*atp*H) between 385 individuals from 47 populations from the QTP and adjacent areas. Our objectives were: (1) to elucidate the intraspecific divergence of *H. vulgaris* in relation to the Quaternary climatic fluctuations, and, (2) to infer historical population range expansion of this aquatic plant on the QTP.

## Materials and Methods

### Ethics Statement

This study was conducted in accordance with all People's Republic of China laws. No specific permits were required for the described field studies. No specific permissions were required for access to the locations described in this study. The location is not privately owned and neither is it protected in any way. This study species is also not protected by any law.

### Plant sampling

**Figure 1:**
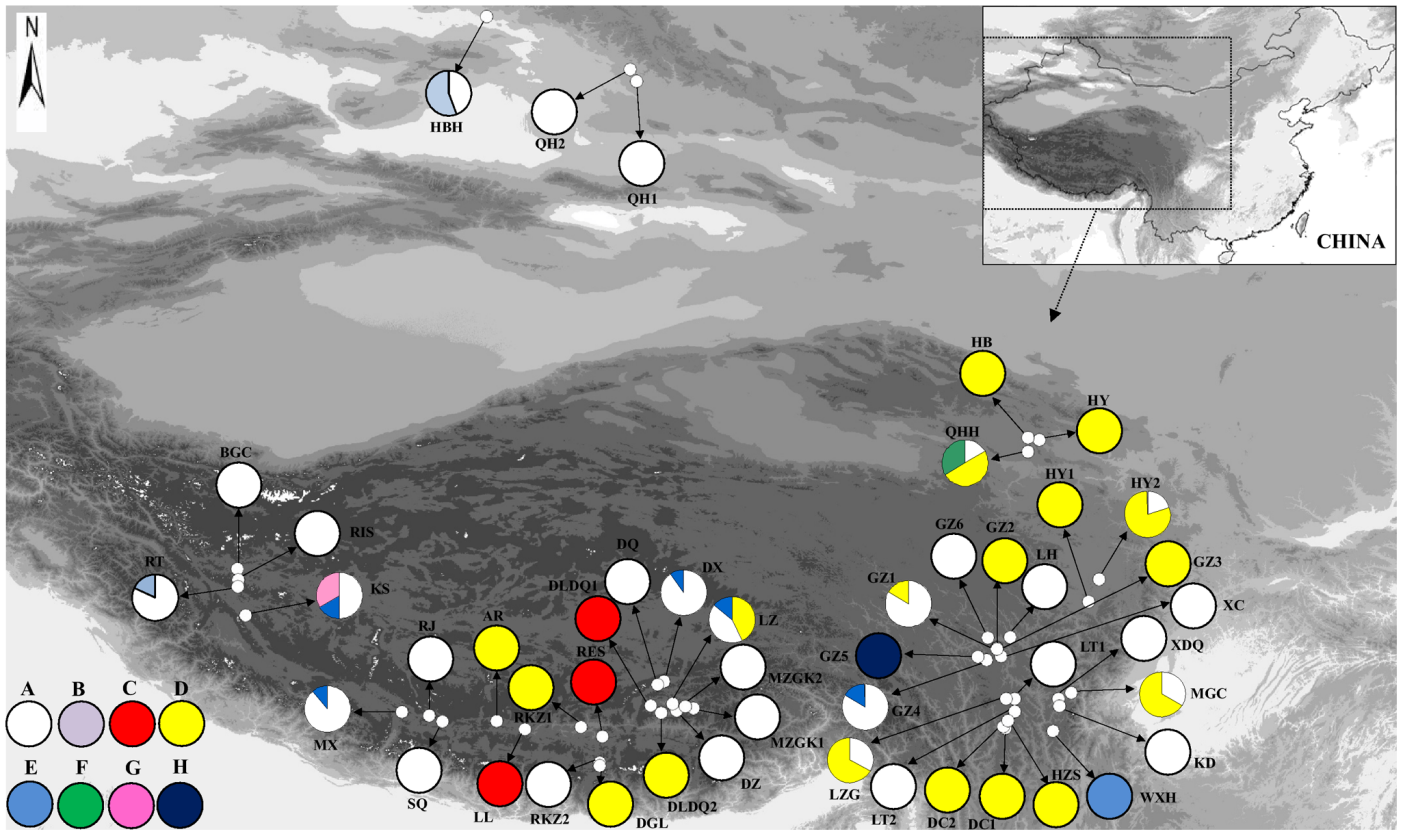
Collection localities (population codes as in Table 1) and the geographical distributions of eight chloroplast haplotypes (A–H) found in 47 populations of *H*. *vulgaris* in the Qinghai-Tibetan Plateau and neighboring areas.

Leaf material from each individual of *H*. *vulgaris* was collected from populations across the QTP and neighboring areas, from the southeast (the HDM) to the northeast (Qinghai province) to the north (Xinjiang province) (Fig. 1). The sampled individuals within populations were at least 3 m apart by distance. Collected fresh leaves were dried immediately with silica gel and stored at room temperature. In total, 385 individuals from 47 populations were included in the study. Details on the collection sites including the latitude, longitude, altitude and the sample size are provided in Table 1. Voucher specimens from all populations were deposited in the Wuhan Botanical Garden (WBG) herbarium.

**Table 1:** Details of sample locations, samples size (*N*), haplotypes and haplotype diversity (*h*) of 47 populations of *Hippuris vulgaris* surveyed for DNA sequence variation at four combined chloroplast regions.

Population code	Sample location	Coordinates (E/N)	Altitude (m)	*N*	Haplotypes	*h*
HBH	Habahe, Xinjiang	86°24′/48°04′	535	9	A,B	0.556
QH1	Qinghe, Xinjiang	90°22′/46°39′	1210	8	A	0.000
QH2	Qinghe, Xinjiang	90°24′/46°40′	1211	8	A	0.000
HY	Huangyuan, Qinghai	101°02′/36°52′	2979	8	D	0.000
HB	Haibei, Qinghai	100°43′/36°56′	3048	5	D	0.000
QHH	Qinghaihu, Qinghai	100°21′/36°42′	3100	6	A,D,F	0.733
MZGK1	Mozhugongka, Xizang	91°52′/29°46′	3895	7	A	0.000
DLDQ1	Duilongdeqing, Xizang	90°45′/29°50′	3794	9	C	0.000
DX	Dangxiong, Xizang	91°06′/30°29′	4724	10	A,E	0.200
DZ	Dazi, Xizang	91°26′/29°42′	3677	8	A	0.000
DQ	Deqing, Xizang	90°56′/30°23′	4176	7	A	0.000
RKZ1	Rikaze, Xizang	88°54′/29°16′	3816	6	D	0.000
MZGK2	Mozhugongka, Xizang	91°39′/29°48′	3740	8	A	0.000
LZ	Linzhou, Xizang	91°19′/29°53′	3712	7	A,D,E	0.714
DLDQ2	Duilongdeqing, Xizang	91°01′/29°39′	3642	7	D	0.000
MX	Maxiong, Xizang	84°09′/29°41′	4540	9	A,E	0.222
RKZ2	Rikaze, Xizang	89°24′/28°20′	4446	8	A	0.000
SQ	Shaqu, Xizang	85°14′/29°25′	4679	8	A	0.000
RJ	Rujiao, Xizang	84°54′/29°34′	4578	8	A	0.000
DGL	Donggala, Xizang	89°23′/28°14′	4466	8	D	0.000
KS	Kunsha, Xizang	80°02′/32°13′	4233	6	A,E,G	0.733
BGC	Bangongcuo, Xizang	79°49′/33°27′	4266	8	A	0.000
RIS	Risong, Xizang	79°50′/33°10′	4307	8	A	0.000
RES	Resuo, Xizang	89°28′/29°01′	3951	8	C	0.000
RT	Ritu, Xizang	79°49′/32°59′	4342	11	A,B	0.327
LL	Lulong, Xizang	87°25′/29°13′	4299	8	C	0.000
AR	Angren, Xizang	86°40′/29°26′	4557	8	D	0.000
XDQ	Xinduqiao, Sichuan	101°32′/30°01′	3464	8	A	0.000
WXH	Wuxuhai, Sichuan	101°24′/29°10′	3706	8	E	0.000
LT1	Litang, Sichuan	100°23′/30°01′	4019	16	A	0.000
KD	Kangding, Sichuan	101°33′/29°50′	3353	8	A	0.000
MGC	Mugecuo, Sichuan	101°52′/30°11′	2600	6	A,D	0.533
LZG	Luozigu, Sichuan	100°09′/30°01′	3960	6	A,D	0.533
LT2	Litang, Sichuan	100°23′/29°41′	3671	8	A	0.000
DC1	Daocheng, Sichuan	100°05′/29°14′	3987	8	D	0.000
DC2	Daocheng, Sichuan	100°05′/29°17′	4089	16	D	0.000
HZS	Haizishan, Sichuan	100°11′/29°26′	4649	8	D	0.000
XC	Xiangcheng, Sichuan	100°03′/29°08′	4614	8	A	0.000
GZ1	Ganzi, Sichuan	99°55′/31°22′	4243	6	A,D	0.333
GZ2	Ganzi, Sichuan	99°55′/31°21′	4281	8	D	0.000
GZ3	Ganzi, Sichuan	99°55′/31°20′	4118	8	D	0.000
GZ4	Ganzi, Sichuan	99°38′/31°02′	3982	6	A,E	0.333
GZ5	Ganzi, Sichuan	99°24′/31°08′	3995	8	H	0.000
GZ6	Ganzi, Sichuan	99°40′/31°38′	3908	16	A	0.000
LH	Luhuo, Sichuan	100°16′/31°39′	3502	8	A	0.000
HY1	Hongyuan, Sichuan	102°20′/32°35′	3529	8	D	0.000
HY2	Hongyuan, Sichuan	102°37′/33°10′	3466	6	A,D	0.333

### DNA extraction, PCR amplification and sequencing

Total genomic DNA was isolated from 0.5 g of silica-dried leaf tissue following the CTAB method [51]. After preliminary screening of 20 non-coding regions of cpDNA among six individuals from six geographically distant populations of *H*. *vulgaris*, polymorphism was observed in four regions, *ycf*6-*psb*M [52], *trn*T-*trn*L [53], *rps*16 intron [52] and *atp*I-*atp*H [54]. These four cpDNA fragments were amplified and sequenced in all individuals sampled. PCR reactions were carried out in a volume of 50 µl containing 0.25 mM of each dNTP, 5 µl of 10×Taq buffer [10 mM Tris-HCl (pH 8.3), 1.5 mM MgCl_2_, and 50 mM KCl], 1 mM of each primer, 2 U Taq Polymerase (TaKaRa, Dalian, China), and 60 ng of DNA template. Amplification of genomic DNA was carried out on a PTC-100™ thermocycler (Bio-Rad, Hercules, CA, USA). It was commenced with 5 min at 94°C and annealing at 60°C for 5 min, followed by 30 cycles of 1 min at 94°C, 2 min annealing at 50–60°C (depending on the kinds of primers), and 2 min extension at 72 °C, and a final extension cycle of 10 min at 72°C.

The size of PCR products was determined electrophoretically on 1.5% (W/V) agarose gels run at 100 V in 0.5×TBE (Tris-boric acid-EDTA) and visualized by staining with ethidium bromide. All PCR products were purified from agarose gel using the TIANquick Midi Purification Kit following the protocols provided by the manufacturer (Tiangen, Beijing, China). The purified PCR products were sequenced using the ABI Prism BigDye terminator cycle sequencing ready reaction kit (Applied Biosystems, Foster City, CA, USA) and performed on an ABI 3730 automated sequencer (Applied Biosystems, Foster City, CA, USA).

### Phylogenetic analysis

CpDNA sequences were aligned by the program Clustal W [55] and then adjusted manually. Insertions/deletions (indels) were firstly treated as a single character resulting from one mutation. CpDNA haplotypes were determined from nucleotide substitutions and indels of the aligned sequences of *ycf*6-*psb*M, *trn*T-*trn*L, the *rps*16 intron, and *atp*I-*atp*H. For a comparison, the cpDNA haplotypes were also determined from the substitutions only. Phylogenetic analyses of cpDNA haplotype sequences were performed by the neighbor-joining (NJ) method. The NJ tree was constructed according to Kimura's two-parameter model [56] using MEGA 3 [57]. The mid-point rooting method which assuming approximately equal evolutionary rates over all the branches was used to root the NJ tree. Confidence values at the nodes were tested by performing 1,000 bootstrap replicates [58]. Phylogenetic analyses of cpDNA haplotype sequences were also reconstructed using a Bayesian approach. The best-fit model of DNA substitutions (F81) was selected with the Akaike information criterion (AIC) in MrModeltest v2.3 [59]. The Bayesian Markov Chain Monte Carlo (BMCMC) estimate of phylogeny was inferred using MrBayes v. 3.1.2 [60] with default priors. Bayesian analysis was performed for 10,000,000 generations. Two simultaneous chains were run with sampling every 1,000 generations. Tracer v1.5 [61] was used to check the likelihood scores of sampled trees. The first 25% trees were discarded and > 50% posterior probability consensus trees from the remaining trees were calculated. Pairwise differences between DNA haplotypes were calculated using ARLEQUIN ver. 3.1 [62]. A statistical parsimony haplotype network based on the matrix of pairwise differences between DNA haplotypes was obtained with the aid of the TCS 1.06 [63] using the 95% connection probability limit and treating gaps as single evolutionary events.

### Population and phylogeographic analyses

To measure levels of genetic variation, haplotype diversity (*h*) and nucleotide diversity (*pi*) for each population and for the entire species were calculated using ARLEQUIN ver. 3.1 [62]. The average gene diversity within populations (*H*_S_), total gene diversity (*H*_T_), proportion of total diversity caused by differences between populations (*G*_ST_), and the number of substitution types (*N*_ST_) were estimated from cpDNA haplotypes following the methods described by Pons and Petit [64] using the program PERMUT (available at http://www.pierroton.inra.fr/genetics/labo/Software/Permut/; 1000 permutations test). A comparison was made between *G*_ST_ and *N*_ST_ using the U-statistic, which is approximated by a Gaussian variable taking into account the covariance between *G*_ST_ and *N*_ST_. *G*_ST_ depends only on the haplotype frequency, whereas *N*_ST_ takes into account differences between haplotypes. If *N*_ST_ is significantly higher than *G*_ST_, closely related haplotypes occur more often in the same populations than less closely related haplotypes, indicating the presence of phylogeographical structure [64].

The spatial genetic structure of haplotypes was analyzed by spatial analysis of molecular variance (SAMOVA) using SAMOVA v. 1.0 [65]. SAMOVA iteratively seeks the composition of a user-defined number (*K*) of groups of geographically adjacent populations that maximizes *F*_CT_, i.e., differences among groups of populations. The most likely number of groups (*K*) was determined by repeatedly running SAMOVA with 2–10 groups and choosing those partitions with a maximum *F*_CT_ value, as suggested by Dupanloup et al. [65]. For each *K*, the configuration with the largest *F*_CT_ value after the 100 repetitions was retained as the best grouping of populations. Hierarchical analysis of molecular variance (AMOVA; [66]) was also conducted to quantify the proportion of total genetic variance explained by differences between regional population groups (as identified by SAMOVA) and between populations within groups. The AMOVA was performed with the program ARLEQUIN ver. 3.1 [62] and significance of variance components were tested with 10, 000 permutations.

The *F* statistic (*F*_ST_) between populations was calculated using the program ARLEQUIN ver. 3.1 [62], and 1,000 permutations were used for significance testing in all cases. To test the significance of isolation by distance between populations, the Mantel test with 1,000 random permutations on matrices of pairwise population *F*_ST_ values and the geographical distances was performed using ARLEQUIN ver. 3.1 [62].

To infer the historic demographic expansion events within each inferred haplotype clade, we carried out mismatch distribution analyses in ARLEQUIN ver. 3.1 [62]. The goodness-of-fit under a sudden-expansion model was tested with the sum of squared deviations (SSD) and Harpending's raggedness index (Rag) [67]. A parametric bootstrap approach [68] with 1,000 replicates was used to test the observed mismatch distribution's fit to the sudden expansion model. If the sudden-expansion model was not rejected, the expansion parameter (τ) was converted to an estimate of time (*T*, in number of generations) since expansion began using *T*  = τ/2*u* [69], [70], where τ is the mode of the mismatch distribution and *u* is the neutral mutation rate for the entire sequence per generation. The value for *u* was calculated as *u* = *µkg*, where *µ* is the substitution rate in substitutions per site per year (s/s/y), *k* is the average sequence length of the DNA region under study (each indel was treated as one mutation) and *g* is the generation time in years (i.e. the average age at which reproduction first occurs). Rate-constancy of cpDNA haplotype evolution in *H*. *vulgaris* was evaluated by relative rate tests in MEGA 3 [57]. After discovery of rate-constancy, the average cpDNA mutation rate of 1.52×10^−9^ substitutions per site per year (s/s/y) [71] was used to estimate the expansion time. This substitution rate value used here is approximate estimate but fall within the range commonly reported for non-coding chloroplast regions of seed plants [72], [73]. The generation time of *H*. *vulgaris* was assumed to be one year (J.M. Chen, pers. obs.).

### Divergence time between lineages

A Bayesian approach was used to estimate the divergent times for different *H*. *vulgaris* lineages using BEAST v 1.5.4 [74]. CpDNA haplotype sequences were found to follow the GTR+Γ+G model as inferred from MrModeltest v. 2.3 [59] with six Gamma categories; a starting tree was randomly generated and a Yule process was performed prior. We assumed an evolutionary rate of 1.52×10^−9^ s/s/y [71] for the four combined chloroplast non-coding regions. This rate was then used for estimating lineage divergence time in BEAST under an uncorrelated lognormal relaxed molecular clock assumption. Two separate MCMC analyses were run for 10,000,000 generations with sampling at every 1,000 generations to ensure all the effective sample size (ESS) values greater than 200. Tracer v1.5 [61] was used to check the parameters and the first 25% generations were discarded as burn-in.

## Results

### Chloroplast DNA diversity

**Figure 2:**
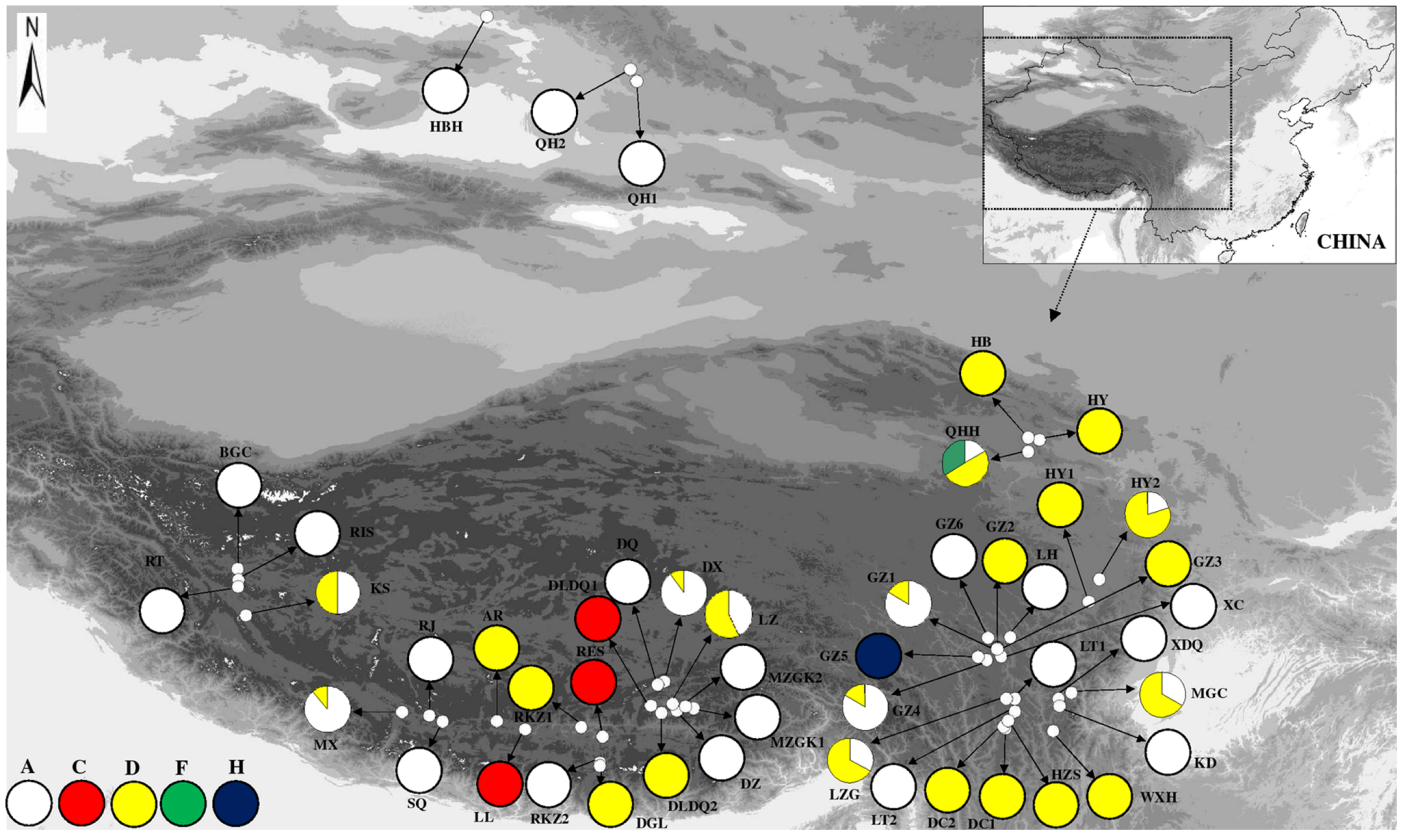
The geographical distributions of the five chloroplast haplotypes (A, C, D, F and H) based on substitutions in only 47 populations of *H*. *vulgaris* in the Qinghai-Tibetan Plateau and neighboring areas.

The aligned sequences of *ycf*6-*psb*M, *trn*T-*trn*L, the *rps*16 intron and *atp*I-*atp*H were 515, 427, 513, and 651 bp in length, respectively. The total length of combined alignments was 2,106 bp. A total of eight polymorphic sites (four substitutions and four indels) of the combined four cpDNA non-coding regions resulted in the resolution of eight haplotypes (A–H) across the 385 individuals (47 populations) of *H*. *vulgaris* sampled (Table 1 and Table 2). GenBank accession number (Nos. JX415319 to JX425330) for each of the cpDNA non-coding region of each chloroplast haplotype is listed in Table S1. At the species level, the estimated haplotype diversity was *h* = 0.604. Haplotype diversity varied across populations, ranging from 0.000 to 0.733, the QHH and KS populations have the highest *h* values (Table 1). Nucleotide diversity was estimated within the species as a whole (*pi* = 0.0020±0.0010) and within populations, ranging from 0.0000 to 0.2167, the QHH population has the highest *pi* value (data not shown). Within-population gene diversity (*H*_S_) was 0.111 and total gene diversity (*H*_T_) was 0.615. Parameters of *V*_S_ and *V*_T_ were 0.071 and 0.484, separately. From the 47 populations sampled, 35 were fixed for a single chlorotype and only 12 populations were polymorphic with two or three haplotypes (Fig. 1, Table 1). Among the eight cpDNA haplotypes revealed in *H*. *vulgaris* in this study, haplotypes A and D were found to be the most widespread. Thirty of the 47 populations investigated harbored haplotype A and 18 populations harbored haplotype D. The haplotype E was found in five populations (Fig. 1, Table 1). When the indels were excluded, there were only five haplotypes remained (A, C, D, F and H) (Table 2). The distribution pattern of these haplotypes was similar to that of haplotypes based on substitutes and indels, e.g., haplotypes A and D were found to be widely distributed in the studied populations; 10 populations harbored two or three haplotypes, and haplotypes F and H were unique to a particular population, separately (Fig. 2). Thus, the following analyses of population structure and phylogeny were based only on the haplotypes realized from substitutes and indels.

**Table 2:** Chloroplast DNA sequence polymorphism detected in four non-coding regions of *Hippuris vulgaris* identifying eight haplotypes (A–H).

Haplotypes	Variable sites							
	*ycf*6-*psb*M		*trn*T-*trn*L			*rps*16 intron		*atp*I-*atp*H
	1	3			3	3	3	3
	5	2		6	6	0	4	0
	9	6	9	0	8	4	2	0
A	T	-	-	-	-	G	T	A
B	.	.	#	.	.	.	.	.
C	.	.	.	.	§	A	.	.
D	.	<$>\raster="rg1"<$>	.	.	.	.	.	C
E	.	.	.	.	.	.	.	C
F	G	.	.	.	.	.	.	.
G	.	.	#	⊚	.	.	.	.
H	.	<$>\raster="rg1"<$>	.	.	.	.	G	C

-: alignment gap; <$>\raster="rg1"<$>: TATAT; #: TAGAACCG; ⊚: TATTTG; §: TGTCATG.

### Population structure

The test for phylogeographical structure in the variation of cpDNA haplotype showed that

*N*_ST_ was not significantly higher than *G*_ST_ (0.853 and 0.819, respectively) indicating no phylogeographic structure. In the SAMOVA analyses, *F*_CT_ values increased progressively as the value of *K* was increased from 2 to 10, which did not allow us to unambiguously identify the number *K* of groups of populations. The Mantel test revealed no significant correlation between genetic and geographical distances of cpDNA haplotypes (*r* = −0.01582, *P* = 0.575) over all populations.

### Phylogenetic relationships and geographical distribution of haplotypes

**Figure 3:**
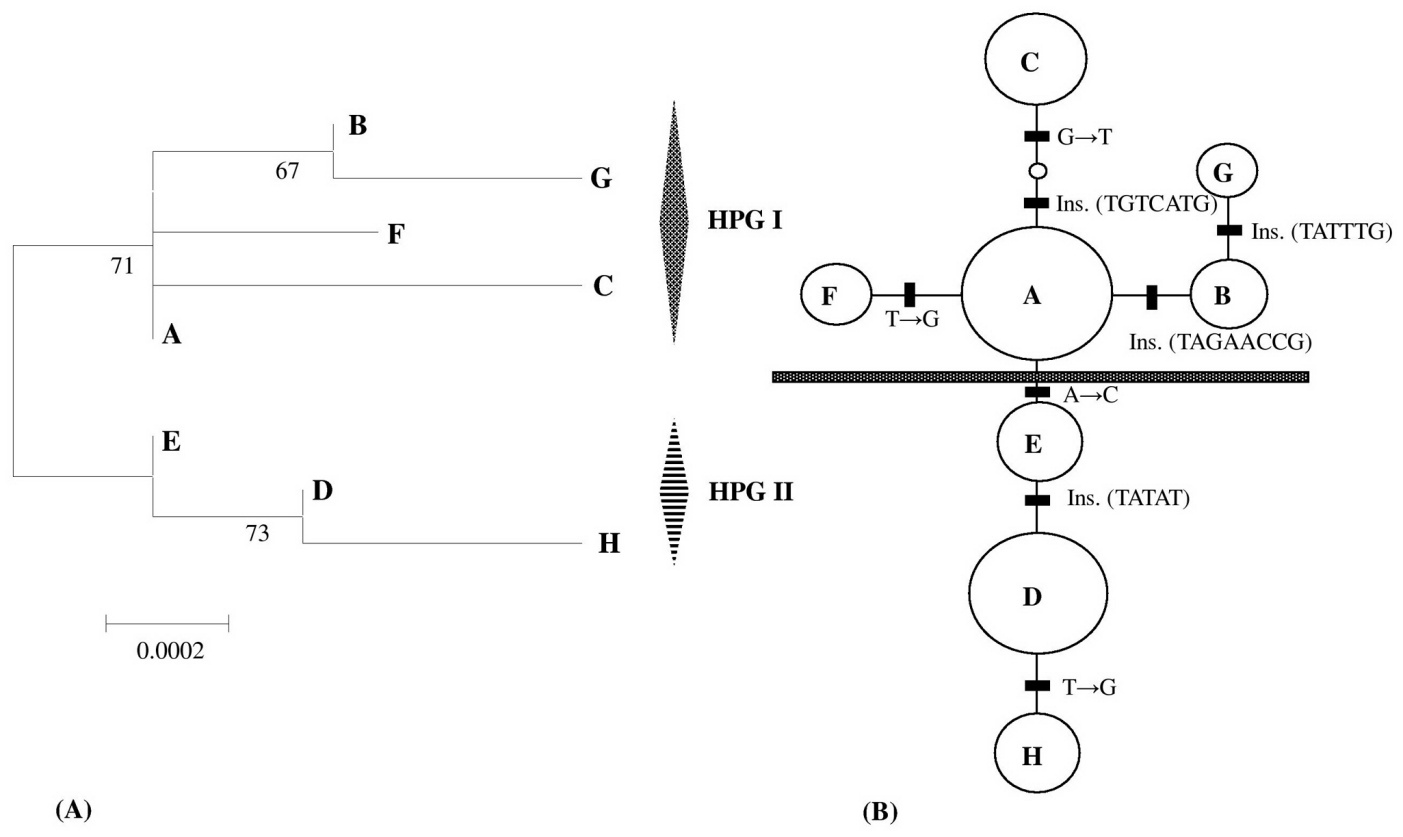
Phylogeny of the eight chloroplast haplotypes (A–H) detected in *H*. *vulgaris*. (**A**) Neighbor-joining clustering of the eight chloroplast haplotypes. Numbers above branches indicate the bootstrap values (>50% are shown) for NJ (Left, 1,000 replicates) and Bayesian analyses (Right). HPG: Haplogroup; (**B**) 95% plausible network of the eight chloroplast haplotypes. Each solid line represents one mutational step that interconnects two haplotypes for which parsimony is supported at the 95% level. The distribution of a certain haplotype is marked in the circles. The small open circle indicates an inferred intermediate haplotype not deteched in this investigation. The size of each circle is proportional to the haplotype frequency.

The neighbor-joining tree of the eight cpDNA haplotypes detected from the 385 individuals of *H*. *vulgari* (Fig. 3A) was identical to the tree reconstructed under a Bayesian approach. The unrooted TCS network of all *H*. *vulgaris* haplotypes (Fig. 3B) is consistent with the trees created from the NJ and Bayesian methods. Among these haplotypes, haplotype A was widely distributed in all the sampled regions (30 populations). Haplotype F was unique to population QHH from the east edge of the Plateau and G was confined to population KS from the interior of the Plateau. Haplotype B was found in one population (HBH) from the Xinjiang province and one population (RT) from the interior of the Plateau. Haplotype C was also found in three populations from the interior region (Fig. 1); HPG II contained three haplotypes (D, E and H) (Fig. 3). Among these haplotypes, haplotype D was widely distributed on the east edge of the QTP, e.g., the HDM (10 populations) and all the sampled populations from Qinghai province (3 populations). In this region, haplotype H was unique to population GZ5 and haplotype E was found in two populations (WXH and GZ4). In addition, haplotype D was found in five populations from the interior of the QTP, e.g., Xizang province. Haplotype E was also found in four populations from the interior region (Fig. 1).

### Demographic analyses

Mismatch analyses indicated that the distribution of pairwise differences for HPG I and HPG II were unimodal. Both the observed variance (SSD) and the HRag for each of the two groups was not significantly different from that expected under the population expansion model (*P*>0.05), which further supported that HPG I and HPG II had experienced rapid expansions. The expansions for HPG I and HPG II were estimated to have been at 0.12 and 0.17 Myr (Table 3).

**Table 3:** Mismatch distribution analyses and estimation of expansion time.

Groups	Mismatch distribution analyses			
	SSD (*P*_SSD_)	Raggedness index (*P_Ra_*_g_)	τ	Expansion time (Myr)
HGP I	0.074 (0.112)	0.193 (0.170)	1.052	0.12
HGP II	0.053 (0.095)	0.151 (0.138)	0.773	0.17

### Lineages divergence time

**Figure 4:**
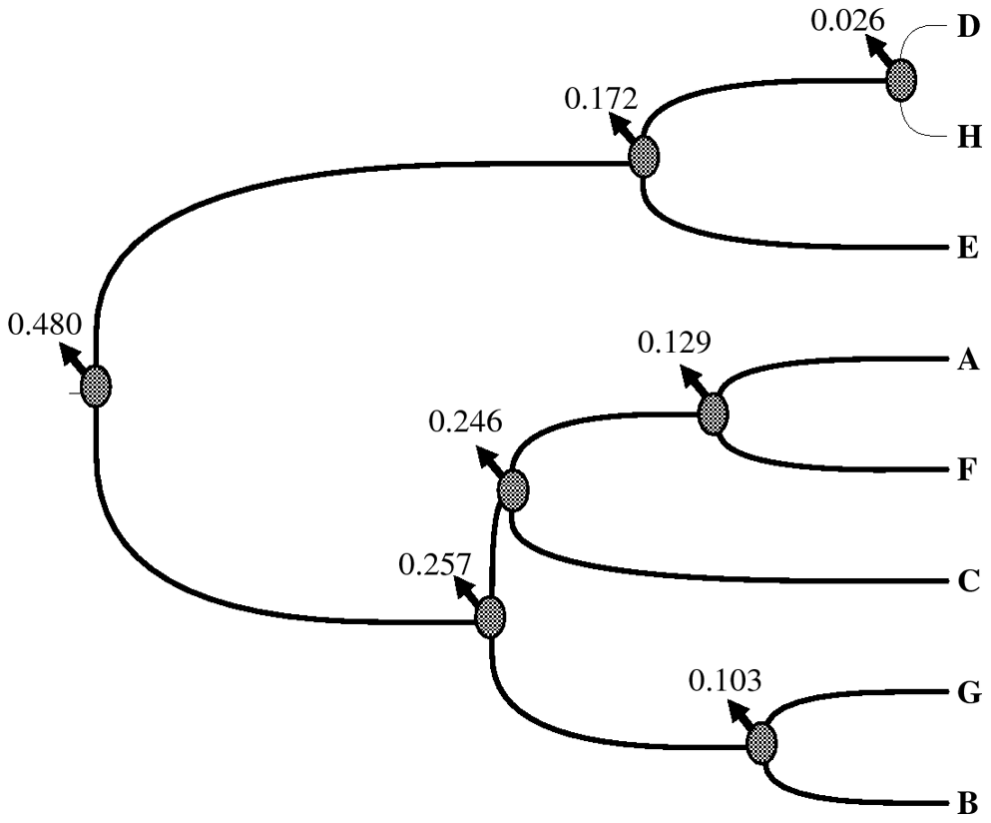
Bayesian analyses of the divergent time (Myr) between chloroplast DNA haplotypes.

The divergence times between the haplotypes estimated by Bayesian method ranged from 0.480 Myr to 0.026 Myr (Fig. 4). The divergence time of the two main groups of haplotypes (HPG I and II) were estimated at 0.480 Myr, which suggests that the divergence among haplotypes of *H*. *vulgaris* falls into mid-to-late Pleistocene.

## Discussion

For the aquatic herb *H*. *vulgaris* from the QTP and adjacent areas, inter-population differentiation was high, with *G*_ST_ = 0.819 occurred among populations, indicating high genetic differentiation across the sampled region. Unlike most previous studies, which show high genetic differentiation coupled with distinct phylogeographical structure [29], [75], the phylogeographical structure among the sampled populations of *H*. *vulgaris* was not obvious. Although two haplotype groups were recognized from the NJ tree, Bayesian methods and the haplotype network, the distributions of haplotype groups did not correspond to distinct geographical areas. SAMOVA analyses also failed to detect any meaningful geographical groups of the sampled populations based on the cpDNA haplotypes. A lack of significant geographical structure of populations was also confirmed by the results of haplotype-identification permutation tests, which indicated that *N*_ST_ was not significantly larger than *G*_ST_ (*N*_ST_ = 0.853>*G*_ST_ = 0.819, *P*>0.05). Lack of significant phylogeographical structure in *H*. *vulgaris* from the QTP and adjacent areas may result mainly from the occurrence of widespread haplotypes, i.e., haplotypes A and D, across the sampled regions. As a widespread, clonal and wind-pollinated herbaceous plant species, *H*. *vulgaris* populations were expected to be less differentiated [76], [77]. However, contrary to the expectation, populations were greatly differentiated although they shared several common haplotypes. The high population differentiation was probably because of the different frequencies of these common haplotypes occurred in each region.

In this study, the divergence times for all the haplotypes were estimated to be less than 0.5 Myr. The genetic divergence between the two intraspecific lineages of the species occurred at 0.48 Myr, corresponding to the mid-to-late Pleistocene [20]. This divergence within the species falls within the most extensive glaciations during the Naynayxungla Glaciation (0.8–0.5 Myr), when there were many large ice caps, glacier complexes and great valley glaciers, covering a total area≥500,000 km^2^ [20]. This extensive glacial advance likely continued until 0.17 Myr after the penultimate glaciations (0.3–0.13 Myr) [20], [21]–[23]. During these glaciations, the ice sheet is thought to have covered an area five to seven times larger than it does today, and even during the interglacial warming periods, the central QTP region retained glaciers [20], [78]–[80]. The glaciers and/or extremely low temperature in the high mountains during these stages might have created barriers to gene flow between geographically isolated populations of *H*. *vulgaris*, which therefore promoted its intraspecific divergence.

For each of the intraspecific lineages, our mismatch distribution analyses showed a unimodal distribution, suggesting a recent range expansion had occurred. Both the observed variance (SSD) and the HRag for each of the two groups was also not significantly different from that expected under the population expansion model. In addition, the haplotype network analysis revealed two “star-like” clusters originating from the ancestral haplotypes A and D, respectively, which also supports range expansion. These two expansions were dated to be 0.12 and 0.17 Myr, respectively, which fell within the interglacial stage between the Penultimate Glaciation and the Last Glaciation [20]. Previous studies showed that glacial retreat has occurred since 0.17 Ma [20], [22]. Our results suggest that *H*. *vulgaris* populations expanded as a consequence of this retreat. The widespread haplotypes A and D in each lineage probably originated from the recent expansions during the last interglacial period. *Hippuris vulgaris* is a clonal aquatic herb; vegetative reproduction of this species is by rhizomes, which is potentially a mode of long distance colonization. The simple haplotype composition (many populations were composed of a single haplotype) might be attributed to founder effects. Most of the haplotypes at tip positions were unique to their particular population. Haplotypes A and D may represent the ancestral genotypes and probably produced other mutational derivatives following recent expansions [81], [82].

The two independent range expansions could have begun from different refugee populations. The phylogeographical pattern of cpDNA haplotypes demonstrates two “star-like” clusters that were the result of haplotypes being linked to a central haplotype, but this relatively simple pattern can be explained by populations preserved in refugia that have experienced population expansion after glaciations [83]. However, no specific refugia areas could be deduced because there was no significant geographical structure of the genetic variation (although several haplotypes were unique to a single population). The HDM range of the southeastern QTP is known as an important glacial refugium of many plants [27]. Based on the aquatic plant species richness of different regions of the QTP and adjacent areas, e.g., the total species number decreasing gradually from the HDM region to the interior and to the northwest regions of the plateau [46], we also supported the hypothesis that the HDM region might be a refugium for aquatic plants and that aquatic plant species on the QTP might have colonized their present-day distribution ranges from this refugium. However, we could not exclude the possibility of *H*. *vulgaris* survival in refugia in the interior QTP, because this region possesses a similar cpDNA haplotype diversity and haplotype uniqueness as the HDM region. Plant species survival throughout the Quaternary on the QTP has also been reported in several previous studies [33]–[39]. Six populations distributed in the central region (e.g., LZ population), northeast region (e.g., QHH population) and also the southeast edge (e.g., MGC, LZG, GZ1 and HY2 populations) of the QTP possessed the two widespread haplotypes A and D indicating the populations' admixture might be a result of two independent range expansions.

In three adjacent populations (HBH, QH1 and QH2) of the QTP, only two haplotypes were found. One is the widespread haplotype A and another is the narrow distributed and newly generated haplotype B, which is shared only by HBH and RT populations. Long distance colonization throughout the QTP and northward to the Northwest region of China might be a possible reason for the occurrence of these two haplotypes in northern region of the QTP, which is consistent with previous findings in other plants, e.g., *Pedicularis longiflora* [32] and *Lepisorus clathratus* [8]. However, these two populations (HBH and RT) currently are separated by the Tarim basin, which contains the vast sandy Taklimakan desert, the driest desert in Asia. This region had already begun aridification by the early Pleistocene; the desert and gobi terrain further expanded and the dry climate continued to develop during the middle Pleistocene [84]. Thus, it seems unlikely that *H*. *vulgaris* had colonized through this wide arid region in Northern China, which would have acted as a barrier to dispersal. It was possible that *H*. *vulgaris* had colonized the northwest region of China through other routes, e.g., from the western or eastern edge of the Tarim basin, but this hypothesis still needs further study, with more samples around the Tarim basin included.

In conclusion, from the phylogeographic analyses of *H*. *vulgaris*, we revealed two independent range expansions in response to the Quaternary climatic oscillations on the QTP and adjacent areas. These two expansions might have begun from different refuge populations from before the LGM, which suggested that the *H*. *vulgaris* had survived the LGM and/or previous glaciers since its origins. These findings also partly support the assumption that there was “no massive ice-sheet on the QTP” [24] at least during LGM of the Quaternary. Although our findings on the aquatic herb *H*. *vulgaris* were basically similar to the results obtained for several terrestrial herbs [31]–[33], [38], [39], our study reveals the range shifts during glacial and interglacial periods of aquatic plants occurring on the QTP for the first time, which improves our understanding of phylogeographical patterns of aquatic plants in the QTP and adjacent areas. Although our methods are able to show only the latest range expansions, it seens probable that expansions also occurred during earlier interglacials. Admixture of populations due to repeated range expansions, therefore, might be an explanation of why no significant phylogeographical structure was found and no specific refugia areas could be deduced. Whether other aquatic plant species also display similar evolutionary histories on the QTP as revealed by *H*. *vulgaris* remains a future research question.

## Supporting Information

Table S1 GenBank accession numbers for each of the four non-coding regions of each chloroplast haplotype (A-H) identified in *Hippuris vulgaris*.
(DOC)
