# Supplementary material for: Chloroplast DNA Phylogeography Reveals Repeated Range Expansion in a Widespread Aquatic Herb Hippuris vulgaris in the Qinghai-Tibetan Plateau and Adjacent Areas
Source: PLoS One. 2013 Apr 2;8(4):e60948. doi: 10.1371/journal.pone.0060948 (PMC3614902; doi:10.1371/journal.pone.0060948)
Supplement: Table S1 — GenBank accession numbers for each of the four non-coding regions of each chloroplast haplotype (A-H) identified in Hippuris vulgaris. (DOC) [file pone.0060948.s001.doc]

**Table S1** GenBank accession numbers for each of the four non-coding regions of each chloroplast haplotype (A-H) identified in *Hippuris vulgaris*.

| Haplotype | Regions | | | |
| --- | --- | --- | --- | --- |
|  | *ycf*6-*psb*M | *trn*T-*trn*L | *rps*16 intron | *atp*I*-atp*H |
| A | JX415321 | JX415324 | JX415328 | JX415319 |
| B | JX415321 | JX415325 | JX415328 | JX415319 |
| C | JX415321 | JX415326 | JX415329 | JX415319 |
| D | JX415322 | JX415324 | JX415328 | JX415320 |
| E | JX415321 | JX415324 | JX415328 | JX415320 |
| F | JX415323 | JX415324 | JX415328 | JX415319 |
| G | JX415321 | JX415327 | JX415328 | JX415319 |
| H | JX415322 | JX415324 | JX415330 | JX415320 |
